# Role of Exosomes in Central Nervous System Diseases

**DOI:** 10.3389/fnmol.2019.00240

**Published:** 2019-10-04

**Authors:** Wanying Liu, Xiaodan Bai, Ao Zhang, Juanjuan Huang, Shixin Xu, Junping Zhang

**Affiliations:** ^1^First Teaching Hospital of Tianjin University of Traditional Chinese Medicine, Tianjin, China; ^2^Tianjin Key Laboratory of Translational Research of TCM Prescription and Syndrome, Tianjin, China; ^3^Tianjin University of Traditional Chinese Medicine, Tianjin, China; ^4^Epidemiology, College of Global Public Health, New York University, New York, NY, United States

**Keywords:** exosomes, central nervous system diseases, biomarkers, intercellular communication, stroke

## Abstract

There are many types of intercellular communication, and extracellular vesicles are one of the important forms of this. They are released by a variety of cell types, are heterogeneous, and can roughly be divided into microvesicles and exosomes according to their occurrence and function. Of course, exosomes do not just play a role in cell-to-cell communication. In the nervous system, exosomes can participate in intercellular communication, maintain the myelin sheath, and eliminate waste. Similarly, exosomes in the brain can play a role in central nervous system diseases, such as stroke, Alzheimer’s disease (AD), Parkinson’s disease (PD), prion disease, and traumatic encephalopathy (CTE), with both positive and negative effects (such as the transfer of misfolded proteins). Exosomes contain a variety of key bioactive substances and can therefore be considered as a snapshot of the intracellular environment. Studies have shown that exosomes from the central nervous system can be found in cerebrospinal fluid and peripheral body fluids, and that their contents will change with disease occurrence. Because exosomes can penetrate the blood brain barrier (BBB) and are highly stable in peripheral circulation, they can protect disease-related molecules well and therefore, using exosomes as a biomarker of central nervous system diseases is an attractive prospect as they can be used to monitor disease development and enable early diagnosis and treatment optimization. In this review, we discuss the current state of knowledge of exosomes, and introduce their pathophysiological roles in different diseases of the central nervous system as well as their roles and applications as a viable pathological biomarker.

## Introduction

In the early 1980s, the term “exosome” was used to describe vesicles released by various types of cultured cells with a diameter of 40 to 1000 nm. Over the next few years, the term was refined to describe vesicles originating from the endosome with a diameter between 30 and 100 nm (Luarte et al., 2016; Malm et al., 2016; Yuyama and Igarashi, 2016; Zagrean et al., 2018). According to most of the current literature, vesicular bodies with a bilayer membrane structure which are detached from the cell membrane or secreted by the cells, are called extracellular vesicles (EV). EVs are highly heterologous, they can come from several cell types, are influenced by their microenvironment, and can be detected in various human secretions. All extracellular vesicles are formed by a shared mechanism and are thought to have a common role in cell-to-cell communication by promoting cellular exchange of proteins, DNA, and RNA (Malm et al., 2016; Osier et al., 2018).

**FIGURE 1:**
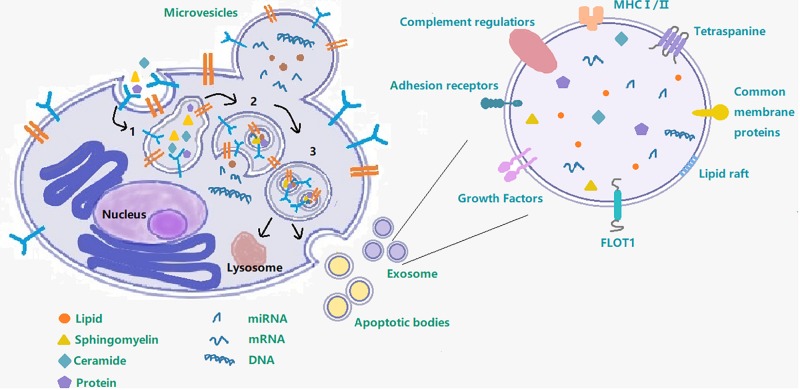
Exosome– cargo, surface markers and endosomal pathway. This figure shows the process of exosome production and secretion. 1 Early endosome; 2 Late endosome; 3 Multivesicular body; following on, the multivesicular body will either fuse with the cell membrane to secrete exosomes out of the cell or fuse with lysosome.

Although exosomes are a subtype of extracellular vesicles (EVs) defined specifically by their diameter (Malm et al., 2016; Yuyama and Igarashi, 2016; Mrowczynski et al., 2019), some published studies use the term EVs to refer just to exosomes. EVs refers to the larger group to which exosomes belong and also includes the apoptotic body (measuring 0.5–5 μm, formed by apoptosis), and microvesicles (measuring 0.05–1 μm, created by part budding), while exosomes originate from endosomes (Tian et al., 2018). Early endosomes will be converted to late endosomes when their protein composition changes, upon maturation, intraluminal vesicles (ILVs) begin to constitute in the lumen of the vesicles via enfoldment of the limiting membrane. Then the ILVs sequester cytoplasmic molecules leading to their accumulation within the late endosome, causing the formation of multivesicular bodies (MVB). MVBs can undergo fusion with lysosomes and with the cellular plasma membrane and then release their contents into the extracellular space (Figure 1). Exosomes produced by various cell types are found in bodily fluids, such as blood, cerebrospinal fluid (CSF), saliva, and urine (Malm et al., 2016; Yuyama and Igarashi, 2016; Cesselli et al., 2018; Osier et al., 2018) and contain proteins, lipids, miRNAs, RNA and DNA. It is precisely because of the transfer of these contents that exosomes can affect intercellular communication under various physiological and pathological conditions (Yuyama and Igarashi, 2016). The most commonly used method to extract exosomes is ultracentrifugation (UC) and storage of them for more than 90 days should be at −80°C (Hong et al., 2018).

The theory that EV can transfer RNA between cells was proposed by the team of Ratajczak et al. (2006) (Clemmens and Lambert, 2018; Stahl and Raposo, 2018). All of these studies have made advances in EV-related research and highlighted the important role of EVs in cell biology, physiology [including stem cell maintenance (Samuelson and Vidal-Puig, 2018)], cancer, neuropathology (Osier et al., 2018; Stahl and Raposo, 2018), stroke, and cardiovascular disease (Stahl and Raposo, 2018). Studies have shown that drugs and comorbidities can affect the mechanisms of biogenesis and release of MVBs and exosomes, thus affecting this mode of intercellular communication. EVs can also play an important role in communication between, and within, key metabolic organs (Clemmens and Lambert, 2018; Samuelson and Vidal-Puig, 2018). Their role in neurological diseases is also particularly prominent, extracellular vesicles (i.e., exosomes in the broad sense) are secreted by several cells and present in the CSF, they are involved in signal transduction not only among neural cells but also among hematopoietic cells and in the peripheral nervous system. The main cause of neurodegenerative diseases is the spread of EVs and the relationship between neurophysiology and neurological disorders, centered on EV-mediated communication between nerves and glial cells, is a critical area of discussion and research. The major causative proteins of various neurodegenerative diseases are encapsulated in EVs derived from CSF and there also appears to be a relationship between them (Kawahara and Hanayama, 2018). The characteristics of exosomes and their physiological and pathological effects make them both an ideal biomarker, and a potential drug delivery vehicle (including use as vectors for targeting gene therapy to specific cells (Clemmens and Lambert, 2018; Leone et al., 2018; Stahl and Raposo, 2018).

## Exosomes in Stroke

Stroke is a complex and life-threatening condition (Mirzaei et al., 2018), and one of the leading causes of death and disability worldwide with high mortality rates in both developing and developed countries. There is ample evidence that both genetic and environmental factors are related to the pathophysiology of stroke (Mirzaei et al., 2018). There are two stroke subtypes: ischemic and hemorrhagic, of which ischemic stroke accounts for about 87% of all strokes (Osier et al., 2018). Ischemic stroke is mainly caused by a thromboembolic occlusion or atherosclerosis of a major artery that supplies the brain, leading to cerebral ischemia and hypoxia (Malm et al., 2016; Zhang and Chopp, 2016). Cerebral hemorrhage is the cause of hemorrhagic stroke, which is usually caused by a weakened arterial wall (i.e., an aneurysm). The ischemic stroke lesion area can be divided into two main areas: the infarct core and the penumbra area (Hong et al., 2018). In the treatment of ischemic stroke, the main goal is to restore blood flow as soon as possible after the onset of symptoms. The two main methods for recanalization are intravenous thrombolysis [tissue plasminogen activator has become the only treatment for stroke patients within 4.5 h of onset (Zhang and Chopp, 2016)] and endovascular intracranial thrombectomy, which can extend patient life by rapidly reanalys of occluded blood vessels and re-establishing tissue perfusion within 12 h of onset (Zhang and Chopp, 2016). The window for treatment with these two methods is very narrow (Hong et al., 2018), and since a series of pathological events usually occurs before a stroke, we therefore need to find an appropriate method of stroke management. One of the main problems here is to identify effective biomarkers for diagnosis and monitoring the treatment of stroke patients (Mirzaei et al., 2018).

**FIGURE 2:**
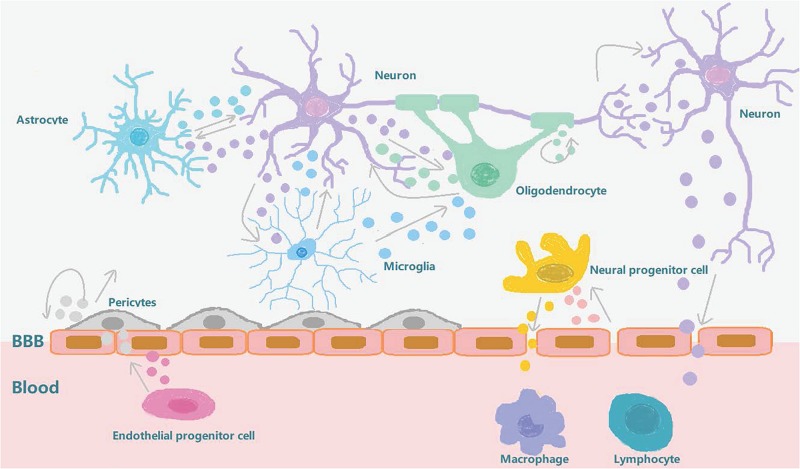
Exosome involvement in brain reconstruction after stroke. After a stroke, the blood-brain barrier is destroyed and the pericytes interact with brain endothelial cells to promote angiogenesis and repair the BBB. The exosomes secreted by the pericytes can also provide nutrition and promote neurogenesis. The exosomes secreted by circulating endothelial progenitor cells can also act on cerebral endothelial cells to promote angiogenesis. Neurons, oligodendrocytes, and astrocytes interact to regulate synaptic plasticity. Neurons interact with microglia to regulate brain immune inflammation, and neurons and neural progenitor cells can secrete exosomes to regulate peripheral immunity after being stimulated by external substances which pass through the BBB. After stroke, brain endothelial cells secrete exosomes that stimulate neural progenitor cells to differentiate into oligodendrocytes to participate in myelination. In conclusion, exosomes can increase long-term neuroprotective effects after stroke, regulate peripheral immune response, and participate in brain reconstruction events such as enhanced angiogenesis and axonal dendritic remodeling.

Biomarkers related to the treatment of diseases, including molecules, proteins, and RNA, have been obtained from a range of bodily fluids (Lausted et al., 2014). These biomarkers have some disadvantages, their low abundance and poor heterogeneity (i.e., it’s not clear which groups these molecules come from), and exosomes have therefore gained more attention since they were discovered, in large part because of their heterogeneity and role in intercellular communication. Stroke recovery is carefully planned by a series of highly interactive processes involving neural stem cells and neurovascular units (Zhang and Chopp, 2016). Emerging data suggests that exosomes play an important role in these processes and mechanisms. After a stroke, exosomes can be synthesized and released from brain cells, and can be detected in peripheral blood or CSF by passing through the BBB. In addition to this, exosomes are also released into the bloodstream from blood cells and endothelial cells in response to stroke (Otero-Ortega et al., 2018). They are involved in increasing long-term neuroprotection after stroke, promoting nerve regeneration, enhancing neurological recovery, and modulating peripheral immune responses. At the same time, they also enhance angiogenesis, neurogenesis, and the remodeling of axon dendrites. The exosomes secreted by brain endothelial cells can also actively participate in brain reconstruction by communicating with brain cells (including neurons and glia) and distal cells in other organs during stroke recovery (Zhang and Chopp, 2016; Figure 2). In addition to beneficial effects, these exosomes can also have adverse effects on distal organs. The release of exosomes from damaged central nervous cells after stroke into the peripheral circulation will affect the spleen, increase the production of circulating pro-inflammatory cytokines, and recruit and activate T and B lymphocytes to regulate peripheral immune inflammatory response. These changes will also affect the heart, kidney, and intestine (Seifert and Offner, 2018; Wang et al., 2019). In the heart, the secretion of inflammatory cytokines by damaged brain cells stimulates the hypothalamus and increases the release of catecholamine, thereby increasing the risk of myocardial ischemia and tumorigenesis (Shoemaker and Goswami, 2015; Venkat et al., 2018). In kidneys, inflammatory mediators induced and secreted after stroke can increase the permeability of renal endothelial cells and make immune cells and inflammatory mediators enter, resulting in renal dysfunction (Wang et al., 2014; Li et al., 2015; Makin et al., 2015). In the intestinal tract, intestinal dysfunction after stroke is characterized by a decrease in the number of beneficial cells in the intestine and an increase in the opportunistic bacteria count. Abnormal bacterial metabolites can also affect the immune response which in turn may adversely affect recovery from stroke (Winek et al., 2016; Arya and Hu, 2018; Zhao et al., 2018; Table 1).

**TABLE 1:** Biomarkers in exosomes associated with stroke.

**Source**	**Contents**	**Mechanism**	**Clinical application**	**References**
Peripheral blood	cystatin *C*, CD14, alpha-lib and talin-1	Vascular event	Early diagnosis	Datta et al., 2014; Kanhai et al., 2014
Serum	caspase-1	Inflammation	Severity judgment	Kerr et al., 2018
Serum	miR-126	Angiogenesis	Severity judgment	Chen et al., 2015; Osier et al., 2018
Plasma	miR-124	Neurogenesis	Early diagnosis	Mishima et al., 2007; Mirzaei et al., 2018
Plasma	miR-30a-5p and miR-21-5p	Inflammation	Early diagnosis, severity judgment and prognosis	Hong et al., 2018; Jiang et al., 2018

*caspase-1, inflammatory protein.*

There are many kinds of exosomes and multiple reports about the proteins they contain, such as cystatin C and CD14, which are involved in the progress of brain atrophy in patients with vascular diseases and whose levels are also associated with the risk of vascular events in patients with coronary artery disease (Kanhai et al., 2014), and integrin alpha-IIb, talin-1 and coagulation cascade proteins which are also associated with vascular events in patients (Datta et al., 2014). Despite the variety and nature of goods contained in exosomes, studies have shown that the main role of the exosome lies in the transmission of related RNA signals, and exosomal miRNAs will vary between different periods of a stroke. MiRNA and RNA networks play major roles in the process of secretion-mediated brain repair and are key mediators in the pathogenesis and pathology of ischemic stroke. One example of this was the study by Collino et al. (2015) whose results showed that when exosomes were released by cells depleted of Drosha protein (producing a total downregulation of miRNAs) the therapeutic effect of the secretion on acute kidney injury was abolished (Luarte et al., 2016). MiRNA has therefore become the most reliable biomarker for the diagnosis, treatment, and prognosis of ischemic stroke. A lot of research on the miRNA and protein content of exosome has been carried out, including Kerr N’s team, who used the multi-analyte automated microfluidic immunoassay platform Plex Assay to detect the levels of caspase-1 inflammatory body proteins in serum and serum-derived EVs from stroke patients. This team has previously shown that inflammatory body proteins are a potential biomarker of CNS damage and also proved that, in the case of brain injury, EV can release inflammatory body proteins into the peripheral circulation. They also located caspase recruitment domains in apoptosis-related proteins (ASC). This can be used as a potential biomarker to help analyze the role of inflammatory bodies in the inflammatory responses following cerebral ischemia (Kerr et al., 2018).

Fan Chen et al. used *in vitro* and *in vivo* methods to study stroke. Wistar rats were exposed to a focal cerebral ischemia model using transient or permanent intraluminal occlusion to the middle cerebral artery (Osier et al., 2018). In *in vitro* culture, compared to DMEM normal controls, miR-126 levels in exosomes from brain endothelial cell were significantly reduced after oxygen-glucose deprivation. In the rat model, compared to levels before ischemic onset, both transient and permanent ischemia samples showed a rapid and significant reduction in exosomal miR-126 at 3 h, and by 24 h, miR-126 levels of exosomes mostly renormalized close to pre-ischemia baseline levels. Serum miR-126 levels were significantly reduced at 3 h of permanent ischemia but did not change significantly after transient ischemia or 24 h. The results showed that the exosomal miR-126 obtained from peripheral blood was more sensitive to the effects of cerebral ischemia and responded to both mild and severe ischemic attacks. However, total serum miR-126 may be a more specific indicator of ischemic severity (Chen et al., 2015; Osier et al., 2018). In another study, the release of miR-124 from brain tissue was considered to play a key role in stroke and there is further evidence that the expression of miR-124 in the central nervous system is 100 times higher than that in other organs and can thus be used as a biomarker (Mishima et al., 2007; Mirzaei et al., 2018). In a study by Wang W’s team, 143 IS patients and 24 non-stroke patients were divided into four phases: hyperacute (HIS), acute (AIS), subacute (SIS), and recovery (RIS), and the expression of miR-30a-5p and miR-21-5p in plasma-derived exosomes in the different stages of ischemic stroke (IS) was examined. The data elucidated that the combination of miR-30a-5p and miR-21-5p can be used to distinguish between HIS, SIS, and RIS, and in the diagnosis of IS. It was hoped that miR-30a-5p might be a favorable biomarker for the diagnosis of HIS, providing a possibility for early clinical diagnosis (Wang W. et al., 2018) although, as Yang et al. pointed out, there are many circulating miRNAs that can be used as diagnostic markers for stroke, and for diagnosis. The results indicated that circulating miR-153, miR-128b, and miR-107, are up-regulated in the stroke group, and so can be used as important biomarkers for diagnosis (Yang et al., 2016). In addition to studies of miRNAs, current studies have expounded the role of other types of ncRNAs, including circular RNAs and long non-coding RNAs, in cerebral hemorrhage (ICH) strokes. These results show that, in the clinic, ncRNAs can be used in the predictive diagnosis of the extent of ICH-induced damage as an optional biomarker (Li et al., 2019).

There are a multiple reports and studies proving that RNA, found in the exosome, has the potential to become a powerful biomarker for stroke diagnosis, treatment, and prognosis. The value of the exosome as a biomarker in the treatment of stroke has also been determined and future research will need to identify specific sources of RNA in circulating and tissue secreted exosomes.

## Neurodegenerative Diseases

Neurodegenerative diseases are one of the major causes of death and disability, and one of the greatest burdens on the health care system (Luarte et al., 2016). Like cerebral apoplexy, exosomes may represent a promising prognostic biomarker and a therapeutic approach to traumatic brain injury and neurodegenerative diseases. Multiple studies have shown that exosomes can initiate and participate in the regulation of neuroinflammation, improve neurogenesis and neurogenic physiological location, and have potential significance for the treatment of certain neurological diseases (Yang Y. et al., 2017; Osier et al., 2018). These neurodegenerative diseases have similar clinical manifestations, but a common feature of a large number of these diseases is the accumulation of insoluble proteins (both extracellular and intracellular) and, as such, these diseases are also called cerebral proteinopathies (Hartmann et al., 2017). For example, AD is characterized by the aggregation of beta-amyloid protein and microtubule-associated protein Tau; PD by the accumulation of neuronal terminal protein alpha-synuclein; ALS by the accumulation of phosphorylated TDP43 (a transcription inhibitor) and superoxide dismutase 1 (SOD1), and Huntington’s disease by the accumulation of mutant Huntington’s protein (mHTT; Luarte et al., 2016).

These neurodegenerative diseases, which present different entities and pathogenesis, have many common features besides the aggregation of erroneously folded proteins. These include; overload of protein clearance pathways, impaired protein homeostasis, and dysfunction or loss of specific neuron populations (Kanata et al., 2018). The neurological dysfunction caused by progressive irreversible degeneration of neurons and synapses is a marker of this kind of disease (Hartmann et al., 2017). It has been shown that many types of cells in the CNS secrete exosomes, including oligodendrocytes, neurons, and astrocytes. Exosomes, as potential carriers, can play a role in the pathogenesis of neurodegenerative diseases and can also help transmission (Wu et al., 2017; Table 2). When released into circulation, they also become a convenient and efficient peripheral non-invasive biomarker for the prediction and diagnosis of various neurodegenerative diseases (Hartmann et al., 2017).

**TABLE 2:** Biomarkers in exosomes associated with neurodegenerative disease.

**Name of disease**	**Source**	**Contents**	**Mechanism**	**Clinical application**	**References**
Alzheimer’s disease	Plasma and CSF	Aβ and NFT	Neuronal damage	Early diagnosis	Wang et al., 2017; Xiao et al., 2017
Alzheimer’s disease	Plasma	REST, HSF-1, Lamp 1 and IRS	Neuronal damage	Early diagnosis	DeLeo and Ikezu, 2018; Pluta and Ulamek-Koziol, 2019
Alzheimer’s disease	Serum	miR-135a, miR-193b and miR-384	Neuronal damage	Early diagnosis	Goetzl et al., 2015b
Parkinson’s disease	CSF	α - SYN, DJ-1 miR-1, miR-485-5p, miR-153, miR-409-	Neuronal damage	Early diagnosis	Joshi et al., 2015; Li et al., 2019
Parkinson’s disease	CSF	3p, miR-433, miR-136-3p, let-7g-3p, miR-19b-3p, miR-10a-5p, miR-132-5p, miR-370 and miR-873-3p	Neuronal damage	Early diagnosis	Yang J. et al., 2017
Prion Diseases	Plasma	PrPSc	Neuronal damage	Early diagnosis	Hartmann et al., 2017
Prion Diseases	Serum	miR-142-3p, miR-143-3p, miR-145a-5p, miR-451a, miR-146a-5p, miR-150-5p, miR-320, miR-let-7b, miR-141-3p, miR-429-3p and miR- 200 family	Neuronal damage and inflammation	Early diagnosis and severity judgment	Reza-Zaldivar et al., 2018; Shah et al., 2018
Amyotrophic lateral sclerosis	Peripheral blood and CSF	TDP-43	Neuronal damage and inflammation	Early diagnosis	Iguchi et al., 2016
Amyotrophic lateral sclerosis	Plasma	miR-183-5p, miR-9-5p, miR-193a-5p and miR- 15a-5p	Neuronal damage	Early diagnosis	Saucier et al., 2019
Huntington’s disease	Plasma	mHtt	Neuronal damage	Early diagnosis and severity judgment	Wang et al., 2017; Denis et al., 2018
Huntington’s disease	Plasma	miR-877-5p, miR-223-3p, miR-30d-5p, miR-128, miR-22-5p, miR-223-5p, miR-222-3p, miR-338-3p, miR-130b-3p_,_ miR-628-3p, miR-361-5p, miR-425-5p	Neuronal damage	Early diagnosis	Kumar et al., 2017

*CSF, cerebrospinal fluid; Aβ plaque, aggregation of beta-amyloid protein; NFT, neurofibrillary tangles; α-SYN, alpha-synuclein; DJ-1, oxidative stress sensor; PrPSc, misfolded prion protein; TDP-43, a protein that helps regulate gene expression; mHtt, produces mutant Huntington protein.*

## Exosomes in Alzheimer’s Disease

Alzheimer’s disease (AD) is the most common neurodegenerative disease, mainly occurring in the form of dementia, which is characterized by severe impairment of cognitive ability, mental state, and ability to carry out activities of daily life. The main pathological markers of AD are the accumulation of Aβ plaque and neurofibrillary tangles (NFT) formed by over-phosphorylation of tau protein. The pathological characteristics of AD can be divided into two types: amyloid plaques and NFTs. The accumulation of Aβ in oligomers (which can lead to amyloid plaque formation) occurs at the earliest stage of disease development (Reza-Zaldivar et al., 2018). Data has shown that about 5.5 million Americans are currently diagnosed with Alzheimer’s disease, and the number is expected to rise to 13.8 million by 2050 (DeLeo and Ikezu, 2018). There is also growing evidence that exosomes are associated with the transmission of Aβ and Tau proteins, but their specific role in the process of Alzheimer’s disease remains controversial. This is because some studies show that exosome transfer of these two proteins can participate in the process of neuronal microtubule decomposition, thus affecting axonal transport, resulting in cell death and neuron loss, but in other studies, exosomes seem to have the ability to reduce brain amyloid beta protein by being ingested by microglia. They can also transmit neuroprotective substances between cells (Malm et al., 2016; Xiao et al., 2017).

Experiments have shown that there are disease-related proteins (Pluta and Ulamek-Koziol, 2019) in exosomes isolated from plasma or CSF samples of patients with AD, which show that exosomes may be used as AD biomarkers. Goetz et al. and Fiandaca et al. found that neuronal exosomes isolated from the plasma of AD patients contained autolysosome proteins, and that LAMP1 levels had changed. Changes in P-tau and Aβ levels were also detected in exosomes isolated from patients’ plasma samples, which proved that they could be used as predictors of AD long before the onset of AD (Malm et al., 2016). Goetzl et al. (2015a, b) and Kapogiannis et al. (2015) also found that there were some neurogenic exosome-related proteins in the plasma of AD patients, including repressor element 1-silencing transcription factor (REST), heat-shock factor 1 (HSF-1), Lamp 1 and phosphorylated type 1 insulin receptor substrate (IRS), and that their levels would change within 10 years prior to the formal diagnosis of AD. They are all therefore likely to be reliable biomarkers for predicting and diagnosing AD. Yang et al. (2018) compared the expression levels of miR-135a, miR-193b, and -384 in serum-derived exosomes of patients with Alzheimer’s dementia (DAT), Parkinson’s disease dementia (PDD), mild cognitive impairment (MCI) and vascular dementia (VaD). Their results showed that compared with the other two small RNAs, microRNA-384 was best able to differentiate AD, PDD and VaD. For the early diagnosis of AD, the combination of mir-384, mir-193b, and mir-135a seems to be more useful (Yang et al., 2018). These experiments not only prove the feasibility of the exosome as a biomarker for the early diagnosis of AD, but also prove that the exosome can provide a new direction for disease identification and prevention.

It seems that exosomes secreted by different brain tissue cells will play different roles in the development and change of AD (Joshi et al., 2015). For soluble Aβ42 species (monomers formed after cutting of amyloid precursors participate in the formation of amyloid plaques), microglia can internalize and degrade them, but they can also secrete exosomes that lead to the aggregation of extracellular soluble Aβ42 species, and neuronal astrocytes, or their exosomes, also promote the aggregation of soluble Aβ42. However, exosomes released by neurons can enhance the ability of microglia to scavenge Aβ42. These findings may be a manifestation of the postsynaptic diffusion mechanism of Aβ in AD (Dinkins et al., 2014; Joshi et al., 2014, 2015). This proves the complexity of the role of exosomes in AD and thus it is essential to further clarify the interaction between different EV populations and different forms of Aβ, and to understand the influence of different EV populations on the diffusion of Aβ assembly between cells to better provide accurate and effective markers for AD and improve the quality of life of patients.

## Exosomes in Parkinson’s Disease

Parkinson’s disease (PD) is the second most common neurodegenerative disease and the most common motor disorder of the central nervous system (CNS) and its incidence increases with aging (Leggio et al., 2017; Zagrean et al., 2018). The main clinical manifestations are; static tremor, muscle stiffness, slow movement, postural instability, and other symptoms caused by dyskinesia. These symptoms are caused by the imbalance between excitatory (acetylcholine) neurotransmitters and inhibitory (DA) neurotransmitters in the substantia nigra of PD patients (Wu et al., 2017). These motor symptoms do not occur immediately after imbalance. The first motor symptoms are observed only when the loss of DAergic neurons in SNpc reaches almost 70% and at least 80% of that DA in the striatum. In addition, PD patients also show a variety of non-motor symptoms, including dementia, and depression (Leggio et al., 2017).

It has been shown that misfolded and aggregated alpha-synuclein (α-syn) is the main component of Lewy bodies and axons in inherited and sporadic forms of PD (Leggio et al., 2017; Tofaris, 2017; Wu et al., 2017). The release of exosomes is closely related to intracellular protein transport along the endosomal-lysosomal pathway, so their biological functions may be related to PD. The cause of PD is also reflected at the molecular level, defective protein transport to endosomes and lysosomes has become a potentially unifying cellular pathway in the pathogenesis of sporadic PD. Because the release of exosomes is closely related to intracellular protein transport along the endosomal-lysosomal pathway, their biology may be relevant to PD (Zagrean et al., 2018).

Studies have shown that exosomes transmit toxic α-syn between cells and induce apoptosis, which is involved in the pathological development of PD. It has also been reported that exosomes have potential neuroprotective effects in PD (Wu et al., 2017). In addition to intercellular communication, exosomes also have the ability to eliminate misfolded proteins which hinder the formation of neural stem cells (Zagrean et al., 2018). Brain neurons and glial cells can also eliminate and reduce harmful metabolites and proteins (e.g., α-syn) in cells by exosome extravasation (Ohmichi et al., 2018). Some studies have shown that exosomes obtained from PD patients can attenuate the neuronal stress response, for example, Tomlinson PR and colleagues performed proteomic analysis of microvesicle preparations from PD patients with inherited and sporadic forms, and added microvesicles to cortical neurons of primary rats deprived of nutrition to observe their biological effects (Tomlinson et al., 2015).

At present, the diagnosis of PD still mainly depends on assessing motor symptoms according to the British Brain Bank Standard and the patient’s response to dopaminergic drugs (Ohmichi et al., 2018). DAergic degeneration before the onset of PD symptoms lasts for about 8–17 years (Leggio et al., 2017), which means that there must be some compensatory mechanism in the early development of PD and therefore, searching for biomarkers of preclinical PD is key to treating and predicting the disease and differentiating it from other diseases with the same manifestations. Because oxidative stress and mitochondrial dysfunction influence the underlying mechanisms of misfolded α-syn aggregation (Picca et al., 2019), biomarkers such as DJ-1 (mitochondrial dysfunction-related) and α-syn have potential as clinical tools for early remission and accurate diagnosis of PD. However, for mitochondrial dysfunction, the main focus is on mitochondrial DNA (mtDNA) analysis with proinflammatory properties (West et al., 2015), as systemic inflammation and mitochondria in the CNS are not known under PD conditions. Whether the disorder is related (Picca et al., 2019). Since there are no defined ranges for α-syn levels in peripheral blood (Ohmichi et al., 2018) no reliable quantitative diagnostic tests for PD have been developed so far. As it is inconvenient to obtain invasive CSF or postmortem (brain tissue) samples to collect these biomarkers (Leggio et al., 2017), the development of specific, non-invasive biochemical markers for the disease is the logical direction to focus research on. Quantitative studies have been carried out on exosomes derived from brain cells in patients with PD to find the best biomarkers, these include neurone-, astrocyte-, and oligodendrocyte-derived exosomes (NDE, ADE, and ODE). One study showed that plasma NDE and ODE levels increased significantly in early stage PD patients and that ODE levels were positively correlated with the severity of the disease. This also demonstrated brain cells’ ability to remove neurotoxic substances (Ohmichi et al., 2018).

With regard to using biomarkers for tracking the progress of PD, Yuki et al. performed protein profiling of plasma-derived exosomes from PD patients at Hoehn and Yahr (HY) stages II and III, and found that in HY stage III PD patients apolipoprotein A1 expression was significantly lower than in HY stage II PD patients. There was no significant difference in fibrinogen gamma chain expression between HY stage II and III PD patients. These results show that the plasma-derived exosome proteins; clusterin, complement C1r subcomponent, and apolipoprotein A1, and the fibrinogen gamma chain, are potential biomarker candidates for PD diagnosis, and exosomal apolipoprotein A1 can be used as a biomarker for tracking PD progression (Kitamura et al., 2018). The feasibility of using peripheral circulating exosomes as biomarkers has been further demonstrated. Recent studies have shown that brain insulin signaling can regulate neuronal survival through the mitogen-activated protein kinase (MAPK) and phosphoinositide 3-kinase-protein kinase B (Akt) downstream pathways, and plays a role in PD pathogenesis. It has also been shown that it is easier to assess time-dependent changes in PD by measuring insulin-signaling markers from brain-derived exosomes than neuroimaging and CSF studies (Athauda et al., 2019). Classical gene mutation is a cause of PD and gene regulation is a topic that attracts people’s attention for study. There are abundant miRNAs in exosomes for diagnosis and treatment, such as miRNA - 124, - 7, - 126, - 155, and so on (Thome et al., 2016; Leggio et al., 2017; Yang J. et al., 2017; Titze-de-Almeida and Titze-de-Almeida, 2018). Gui et al. (2015) found 13 small RNAs in CSF-derived exosomes of PD patients with significant changes, including the KEGG pathway “Dopaminergic synapse,” which was also significantly altered in PD patients with 9 miRNAs (miR-1, -153, -485-5p, -409-3p, -433, -136-3p, -19b-3p, -10a-5p and let-7g-3p) targeting 41 genes in the pathway map of Dopaminergic synapse and the KEGG pathway “Cholinergic synapse” which was significantly enriched in PD patients with 11 miRNAs (miR-1, -153, -132-5p, -485-5p, -409-3p, -433, -370, -873-3p, -19b-3p, -10a-5p and let-7g-3p) targeting 40 genes in the Cholinergic synapse pathway. This was compared with the corresponding levels of miRNAs in AD patients, which proved the reliability of RNA molecules in the exosome as a biomarker and the robustness in distinguishing between PD and AD. These experiments prove that exosomes can be used to help develop new diagnostic and predictive tools.

At present, PD can’t be cured, we can only alleviate the symptoms of patients and minimize the occurrence of dyskinesia. The pre-symptomatic stage of PD emphasizes the importance of early prediction and intervention of the disease (Wu et al., 2017) and it is clear that these experiments also reflect the potential of exosomes for use as biomarkers in AD.

## Exosomes in Prion Diseases

Prion disease, also known as infectious spongiform encephalopathy (TSEs), is characterized by spongiform vacuolation and progressive loss of neuronal structure and function. It is a fatal neurodegenerative disease that occurs mainly in the central and peripheral nervous systems and can be transmitted in animals and humans. The existence of detectable misfolded prion protein (PrPSc) whose main structure is a beta-sheet conformation, is the main pathological feature of prion disease. It is produced by the misfolding of the normal prion protein (PrPC) which is mainly alpha-helical in structure.

PrPC can be highly expressed in exosomes (Hartmann et al., 2017) and disease-related forms of PrPC are also found in exosomes and can be ingested by immature cells. This indicates that exosomes carrying PrPSc can participate in the transmission of viruses between different tissues and promote the development of diseases. In addition, the exosome is also rich in cholesterol, sphingomyelin, and sphingomyelin GPI anchored proteins (which help PrPSc formation), which may also participate in protein sorting in the exosome. Studies have shown that injecting prion-infected exosomes into animals can successfully spread the disease (Cervenakova et al., 2016).

At present, the most well-known transmission routes of prions include cell-cell contact, tunnel nanotubes, and exosomes (Hartmann et al., 2017; Cheng et al., 2018; Heisler et al., 2018). Some studies have shown that PrPSc can come from different cell types, tissues and infection stages, resulting in different mechanisms or combinations of prion transmission, which is not fixed. In other words, there may be multiple prion transmission mechanisms (Guo et al., 2016).

Present research on the role of miRNAs in exosomes with infectious prions is not complete, however, in the late and clinical stages of prion infection, the up-regulation of miR-142-3p, miR-143-3p, miR-145a-5p, miR-451a, miR-146a-5p, miR-150-5p, miR-320 and miR-let-7b, as well as the down-regulation of miR-141-3p, miR-429-3p, all members of the miR-200 family and 182 other miRNA clusters, have all been demonstrated (Boese et al., 2016; Cheng et al., 2018; Shah et al., 2018). The involvement of miRNA in the regulation of complex gene networks may affect various mechanisms of prion disease development, but its key impact points and extent are still unclear.

MiRNAs are valuable as biomarkers of prion diseases, however, further high-throughput studies are needed to identify specific the miRNA(s) in bodily fluids which have potential as biomarkers for the diagnosis and treatment of prion diseases (Kanata et al., 2018). The up- or down-regulation of miRNA in prion-infected exosomes may also be altered in other neurodegenerative diseases, for example, miR-146a-5p, besides being down-regulated in prion diseases, can also be down-regulated in serum of patients with primary progression (PPMS) (Vistbakka et al., 2017), with PD (Wang et al., 2016), and in patients with AD.

Understanding the changes of PrPSc and other neurotoxic proteins *in vivo* and their relationship with exosome release, transport, and related miRNA will help us to manage prion-related diseases from multiple levels (Cheng et al., 2018).

## Exosomes in Amyotrophic Lateral Sclerosis

ALS is a serious neurological disease in which the cortex, brainstem, and spinal motor neurons are affected by prion-like misfolded proteins, e.g., superoxide dismutase-1 (SOD1) and TDP-43, resulting in neurodegeneration and is disease marked by protein dysfunction without RNA or DNA components (Lee et al., 2016; Maguire, 2017). Symptoms include weakness, muscle paralysis, atrophy, and eventually respiratory failure, leading to death. In addition to dysfunction of the motor system, 50% of ALS patients may also have mental illness (Maguire, 2017). The pathophysiology of ALS is complex and there is currently no definite analysis and explanation, nor effective therapy. Most experts believe that its influencing factors may include epigenetics, genetics, stress, poor diet, poor physical fitness, and various environmental factors (Maguire, 2017). Data show that about 5–10% of ALS patients are familial and we can observe important mutations in the SOD1, FUS, TARDBP, C9ORF72, and bone morphogenetic protein modifier genes, all of which increase the susceptibility for ALS, but it has been made clear that the SOD1 mutation is the main genetic factor of ALS (Bonafede et al., 2016; Sohrab et al., 2018). TDP-43 (a protein that helps regulate gene expression) is the main pathological feature of ALS and its level in brain tissue-derived exosomes of ALS patients increases. Central nervous system cells, such as motor neurons, are particularly sensitive to the dysfunction of TDP-43 and incorrect folding of TDP-43 also affects, to some extent, the function of mitochondria (Maguire, 2017).

It is known that exosomes are involved in the transmission of TDP-43 protein and Iguchi et al. believe that, although exosomes contribute to the transmission of TDP-43 proteinosis, they are also a key way to remove TDP-43 aggregates from the neurons and primary neurons of ALS patients. They inhibited the secretion of these exosomes by knocking out the GW4869 or RAB27A gene, which resulted in the aggregation of TDP-43 in the Neuro2a cells of TDP-43A315T transgenic mice. These results showed that the inhibition of exosomes aggravated the disease phenotype of TDP-43A315T mutant transgenic mice, and aggravated cognitive and motor neuron system dysfunction (Iguchi et al., 2016). The exosome has proved to be an important inflammatory mediator of blood mononuclear cells. In ALS, the exosome can carry TDP-43 protein into the external environment and be swallowed by mononuclear cells. Some cytokines, such as IL-6, IL-10, IL-1B and MCP-1, are also secreted reactively under the stimulation of exosomes containing TDP-43 and participate in peripheral immune regulation (Zondler et al., 2017). This illustrates that exosomes associated with ALS exist in the peripheral circulation and interact with other cells and that other substances contained in related exosomes have potential as a biomarker as ALS in addition to TDP-43 protein. As Saucier et al. noted, increased levels of 5 miRNAs were found in plasma-derived exosomes from patients with ALS, levels of 22 miRNAs were reduced, and individual related miRNAs such as miR-183-5p and miR-9-5p, were dysregulated. It has also been shown that miR-193a-5p and miR-15a-5p are associated with ALS progression and can contribute toward diagnosis (Saucier et al., 2019).

## Exosomes in Huntington’s Disease

Huntington’s disease (HD) is a progressive dominant hereditary neurodegenerative disease. It is caused by abnormal amplification of CAG repeats in Huntington’s gene (HTT) and produces mutant Huntington protein (mHTT). It has some neurotoxicity and is one of nine neurodegenerative diseases caused by polyglutamine (polyQ) amplification, however, the mechanism of how mHTT ultimately causes HD remains unclear. It is a great concern that there is no effective means of treatment at present (Zhang et al., 2016; Lee et al., 2017; Wang et al., 2017). Typical symptoms of HD include cognitive impairment and unconscious dance movements (Lee et al., 2017). Although it has been reported that mHTT can be transmitted across nerves through “tunneling nanotubes” and/or vesicle mechanisms, there is no data suggesting that mHTT is directly associated with the exosome during *trans*-nervous transmission (Zhang et al., 2016; Wang et al., 2017).

Zhang and his colleagues used a model culture system to overexpress the HTT-exon 1 polyQ-GFP construct in 293T cells, and showed that EVs contain polyQ-GFP protein and amplified duplicate RNA. Striatal mouse neurons could ingest these EVs, which led to an increase in polyQ-GFP RNA in the cells, although it did not exhibit any significant toxicity during the experimental period. They showed that EVs have the potential to deliver toxic amplified trinucleotide repetitive RNA and so more work needs to be done to assess the fate of the transferred RNA/protein and its potential toxicity to receptor cells. This also seems to indicate that exosomes can act as scavengers, eliminating polyQ proteins from cells to help reduce toxicity. These disease-related amplified RNAs in HD patients’ bodily fluids would seem to be a good choice for finding biomarkers for monitoring disease progression and treatment (Zhang et al., 2016). In another study, statistical analysis revealed that 13 miRNAs were significantly up-regulated in plasma-derived exosomes of HD patients, such as miR-877-5p, miR-223-3p, miR-30d-5p, miR-128, miR-22-5p, miR-223-5p, miR-222-3p, miR-338-3p, miR-130b-3p, miR-628-3p, miR-361-5p, miR-425-5p, and miR-942 (Kumar et al., 2017).

## Exosomes in Traumatic Encephalopathy

Chronic traumatic encephalopathy (CTE) is one of the main causes of death and disability in the world (Xiong et al., 2017). Severe CTE can lead to long-term motor impairment, cognitive impairment and even death, however, the molecular mechanism of neuronal injury and recovery after CTE remains unclear (Huang et al., 2018; Wang and Han, 2019). Currently, the diagnosis of CTE relies on neuroimaging methods, such as magnetic resonance imaging (MRI) and CT scans, and the Glasgow Coma Scale (GCS) (Manek et al., 2018; Cheng et al., 2019). Of these the GCS is the most commonly used, but these diagnostic methods each have their own limitations and difficulties (Manek et al., 2018), which compels us to look for a more reliable diagnostic approach.

The symptoms of CTE can be recovered from in the first year after injury in most cases because about 90% of CTE patients have mild traumatic brain injury. Unfortunately, most patients experience repeated mild traumatic brain injury (mCTE) and thus present with long-term persistent symptoms and even perpetual cognitive dysfunction (Cheng et al., 2019; Karnati et al., 2019). At present, it is difficult to differentiate between, and make a correct diagnosis for, mCTE and CTE (Moyron et al., 2017), but it is certain that neurons, vascular injuries, and inflammatory reactions play key roles in the secondary injury process (Kenney et al., 2018; Ojo et al., 2019), and that the timing of each part is also characteristic (Agoston et al., 2017). There are ongoing efforts to study the pathophysiology mechanism (Manek et al., 2018; Cheng et al., 2019) and, accordingly, obtaining efficient and beneficial biomarkers is essential as they can be used to identify potential pathological changes (Karnati et al., 2019). Promising results have been achieved in the detection of markers from CSF. These biomarkers can be used to predict clinical outcomes, monitor the progression of disease and the persistent pathobiological changes in CTE (Atif and Hicks, 2019) and include markers such as S100β, tau proteins, Neuron-specific enolase (NSE), neurofilament heavy chain protein (NF-H), myelin basic protein (MBP), spectrin breakdown product, glial fibrillary acidic protein (GFAP) and its fragments, and pan C-terminal hydrolase L1 (UCHL1) (Taylor and Gercel-Taylor, 2014; Wang K.K. et al., 2018). However, the biomarkers in these cycles appear very unstable (Kawata et al., 2018; Cheng et al., 2019) and are not very useful in the diagnosis of mCTE.

In addition to these readily soluble proteins obtained by invasive methods, interesting exosomes were later discovered and easily quantified (Kenney et al., 2018). As they are part of intercellular communication mechanisms, they naturally contain a number of pathologically related biologically active substances. Compared to previously found markers, exosomes can better protect the initial state of the contents from hydrolysis and better characterize the time line and severity of CTE due to its two-layer membrane structure (Agoston et al., 2017). In the pathological state, by analyzing the changes in proteomics and genomic profiles (Taylor and Gercel-Taylor, 2014; Karnati et al., 2019) and using cell-specific non-coding RNA (Agoston et al., 2017), we can identify the exosomes (such as form neurons and glial cells) which we need to assess the degree of damage and pathological changes of CTE faster and more accurately. This non-invasive diagnostic approach offers a novel and exciting path for painless diagnosis (Cheng et al., 2019) and opens a new field of study. Studies have indicated that gene expression in saliva-derived exosomes can be used as a biomarker for the diagnosis of mCTE and also demonstrated the existence of a variety of AD-related genes in mCTE patients (Cheng et al., 2019), moreover, exosomes containing Tau are not only a circulating biomarker of AD, they also exist as a biomarker of chronic traumatic encephalopathy after CTE (Ling et al., 2017; Manek et al., 2018). Kenney et al. measured amyloid beta (Aβ), total tau, and phosphorylated tau (p-tau) in plasma and plasma-derived exosomes from 195 veterans (some patients with chronic neuropsychological symptoms). When comparing the results of the mCTE and non-mCTE groups it was found that the exosomes of mCTE soldiers have elevated levels of p-tau and exosome tau, which suggests that blood-derived exosomes have the capacity to be biomarkers of mCTE, and also proved that mCTE is related to chronic neuropsychological symptoms (Kenney et al., 2018). As a promising CTE biomarker, exosomes, if used in conjunction with related physical examinations, will become useful in the early diagnosis and prediction of injury (Karnati et al., 2019).

## Conclusion and Overview

Exosomes can be used as both therapeutic means and ideal biological indicators in central nervous system diseases, and of course, this ability can apply to other diseases as well. The non-invasive nature of exosome analysis means that it can be safely used for prenatal evaluation of fetal central nervous system injury (Goetzl et al., 2019), and diagnostic testing of elderly patients (Cicognola et al., 2019).

Since exosomes released by brain cells can pass through the BBB and enter the peripheral body and CSFs, we should be able to isolate and analyze the cargo contained in these circulating exosomes. This should allow for the isolation of high-efficiency biomarkers, and provide possibilities for monitoring the pathological progress of nervous system diseases and assess treatment effects (Shi et al., 2019). Because exosomes are also modified and targeted, they are also used for treatment (Shi et al., 2019). To make use of these exosomes in the diagnosis of diseases we still need large-scale population studies and their specificity and sensitivity need to be confirmed by further clinical studies. Exosome separation methods and gold standards also needed to be developed to truly distinguish them from contaminating proteins and lipoprotein aggregates. We also need to differentiate exosomes secreted by brain cells to find out which exosome source is the best biomarker for various central nervous system diseases. These issues must be taken into account when aiming to improve the diagnostic value of exosomes (Malm et al., 2016; Wu et al., 2017).

While it is clear that this research area is still at an early stage, especially in the context of the diseases of the central nervous system as described in this review, it can also been seen that the isolation and characterization of exosomes, and the analysis of their contents have attracted considerable attention, causing a surge in recent research output. Further research in this emerging field will have a profound impact on the clinical diagnosis and, over time, treatment of more complex and cryptic diseases.

## Author Contributions

WL provided the idea of this article and wrote it for the first author. XB, AZ, JH, SX, and JZ was responsible for collecting and collating the data.

## Conflict of Interest

The authors declare that the research was conducted in the absence of any commercial or financial relationships that could be construed as a potential conflict of interest.
